# Molecular Allergen-Specific IgE Recognition Profiles and Cumulative Specific IgE Levels Associated with Phenotypes of Cat Allergy

**DOI:** 10.3390/ijms23136984

**Published:** 2022-06-23

**Authors:** Ksenja Riabova, Antonina V. Karsonova, Marianne van Hage, Ulrika Käck, Jon R. Konradsen, Hans Grönlund, Daria Fomina, Evgeny Beltyukov, Polina A. Glazkova, Dmitry Yu. Semenov, Rudolf Valenta, Alexander Karaulov, Mirela Curin

**Affiliations:** 1 Laboratory of Immunopathology, Department of Clinical Immunology and Allergy, Sechenov First Moscow State Medical University, 119991 Moscow, Russia; ryabova_k_a@staff.sechenov.ru (K.R.); karsonova@gmail.com (A.V.K.); daria.s.fomina@gmail.com (D.F.); drkaraulov@mail.ru (A.K.); 2 Division of Immunology and Allergy, Department of Medicine Solna, Karolinska Institutet and Karolinska University Hospital, SE-171 77 Stockholm, Sweden; marianne.van.hage@ki.se; 3 Department of Clinical Science and Education, Södersjukhuset, Karolinska Institutet, SE-171 77 Stockholm, Sweden; ulrika.kack@sll.se; 4 Astrid Lindgren Children’s Hospital, Karolinska University Hospital, SE-171 64 Stockholm, Sweden; jon.konradsen@ki.se; 5 Department of Women’s and Children’s Health, Karolinska Institutet, SE-171 77 Stockholm, Sweden; 6 Therapeutic Immune Design Unit, Department of Clinical Neuroscience, Karolinska Institutet, SE-171 77 Stockholm, Sweden; hans.gronlund@ki.se; 7 Moscow City Center of Allergy and Immunology, Clinical City Hospital #52, 123182 Moscow, Russia; 8 Ural State Medical University, 620014 Ekaterinburg, Russia; asthma@mail.ru; 9 Laboratory of Medical and Physics Research, Moscow Regional Research and Clinical Institute (“MONIKI”), 129110 Moscow, Russia; polinikul@mail.ru; 10 Thoracic Surgery Center, Saint-Petersburg State Research Institute of Phthisiopulmonology of the Ministry of Healthcare of the Russian Federation, 194291 Saint-Petersburg, Russia; semenov_du@mail.ru; 11 Karl Landsteiner University for Healthcare Sciences, 3500 Krems, Austria; 12 Division of Immunopathology, Department of Pathophysiology and Allergy Research, Center for Pathophysiology, Infectiology and Immunology, Medical University of Vienna, 1090 Vienna, Austria; mirelacurin1@gmail.com

**Keywords:** allergy, allergen molecules, cat allergy, cat allergens, IgE reactivity, allergy diagnosis, allergy phenotypes

## Abstract

Cat allergy is a major trigger factor for respiratory reactions (asthma and rhinitis) in patients with immunoglobulin E (IgE) sensitization. In this study, we used a comprehensive panel of purified cat allergen molecules (rFel d 1, nFel d 2, rFel d 3, rFel d 4, rFel d 7, and rFel d 8) that were obtained by recombinant expression in *Escherichia coli* or by purification as natural proteins to study possible associations with different phenotypes of cat allergy (i.e., rhinitis, conjunctivitis, asthma, and dermatitis) by analyzing molecular IgE recognition profiles in a representative cohort of clinically well-characterized adult cat allergic subjects (*n* = 84). IgE levels specific to each of the allergen molecules and to natural cat allergen extract were quantified by ImmunoCAP measurements. Cumulative IgE levels specific to the cat allergen molecules correlated significantly with IgE levels specific to the cat allergen extract, indicating that the panel of allergen molecules resembled IgE epitopes of the natural allergen source. rFel d 1 represented the major cat allergen, which was recognized by 97.2% of cat allergic patients; however, rFel d 3, rFel d 4, and rFel d 7 each showed IgE reactivity in more than 50% of cat allergic patients, indicating the importance of additional allergens in cat allergy. Patients with cat-related skin symptoms showed a trend toward higher IgE levels and/or frequencies of sensitization to each of the tested allergen molecules compared with patients suffering only from rhinitis or asthma, while there were no such differences between patients with rhinitis and asthma. The IgE levels specific to allergen molecules, the IgE levels specific to cat allergen extract, and the IgE levels specific to rFel d 1 were significantly higher in patients with four different symptoms compared with patients with 1–2 symptoms. This difference was more pronounced for the sum of IgE levels specific to the allergen molecules and to cat extract than for IgE levels specific for rFel d 1 alone. Our study indicates that, in addition to rFel d 1, rFel d 3, rFel d 4, and rFel d 7 must be considered as important cat allergens. Furthermore, the cumulative sum of IgE levels specific to cat allergen molecules seems to be a biomarker for identifying patients with complex phenotypes of cat allergy. These findings are important for the diagnosis of IgE sensitization to cats and for the design of allergen-specific immunotherapies for the treatment and prevention of cat allergy.

## 1. Introduction

Furry animals, particularly cats, represent one of the most important indoor allergen sources in most parts of the world [1,2,3,4]. Patients who are allergic to cats suffer from a variety of allergic symptoms, including respiratory symptoms such as rhinitis and asthma, conjunctivitis, and different manifestations of dermatitis (e.g., urticaria and atopic dermatitis) [5,6,7,8,9]. Certain cat allergen molecules such as Fel d 5 and Fel d 6, both of which may contain the carbohydrate galactose-α-1,3-galactose (i.e., α-Gal), and Fel d 2, cat albumin, may show immunoglobulin E (IgE) cross-reactivity with allergens involved in food allergies [10,11,12]. These allergens do not seem to be important in food allergy *per se*, but IgE cross-reactivity needs to be considered in the diagnosis of food allergy [12]. According to the International Union of Immunological Societies (IUIS) allergen nomenclature, several cat allergen molecules have been described [13,14,15,16,17,18,19]. They comprise the major cat allergen Fel d 1, uteroglobin, which was among the first allergens for which the DNA and amino-acid sequences could be determined [20]. In addition, Fel d 2, cat albumin [21]; Fel d 3, cystatin A [22]; Fel d 4 and Fel d 7, two distinct lipocalins [23,24]; and Fel d 8, a latherin-like protein [24], have been identified as cat allergens with IgE-reactive protein epitopes. Fel d 5 and Fel d 6 are cat IgA and IgM, respectively, and contain carbohydrate IgE epitopes of α-Gal, which is of importance in α-Gal-syndrome [25].

Without any doubt, Fel d 1 is the most important cat allergen, showing high IgE reactivity in the majority of cat-sensitized patients and high allergenic activity [26]. It is a major target for IgE antibodies in patients suffering from respiratory cat allergy, and it has been shown that children suffering from cat-related symptoms of asthma are strongly sensitized to Fel d 1 [27]. Accordingly, the development of modern molecular allergen-specific forms of treatment has focused on Fel d 1 [28]. One of these molecular approaches is the use of Fel d 1-derived hypoallergenic T-cell epitope-containing peptides for allergen-specific immunotherapy (AIT), which was evaluated in the first clinical trials using a limited number of Fel d 1 peptides [29]. This approach was then further developed by including additional peptides to achieve broad coverage of MHC class II diversity in patients [30]. With the latter approach, encouraging results were obtained in the first clinical AIT studies performed in an exposure chamber setting, where patients were exposed to cat dander. Unfortunately, the large phase III trial was not successful, which was attributed to a huge placebo effect, which may have been explained by the study design of including cat-exposed individuals who may have developed natural tolerance. A second, more recent allergen-specific treatment approach was based on passive immunization with two Fel d 1-specific monoclonal IgG_4_ antibodies that block the binding of IgE to Fel d 1 in allergic patients [31,32]. The first clinical double-blind, placebo-controlled studies performed in an exposure chamber setting showed that this treatment significantly improved symptoms of cat allergy, which was paralleled by the blocking of IgE recognition of Fel d 1 in treated patients [32].

However, to understand the possible relevance of IgE sensitization to cat allergen molecules other than Fel d 1, it is important to study, as a first step, the IgE sensitization and IgE levels to the complete panel of cat allergen molecules in a representative cohort of clinically well-characterized cat allergic patients. So far, limited data regarding the IgE reactivity of each allergen molecule are available from studies characterizing these allergens, while the full panel has not been studied. Only IgE reactivity to a limited number of cat allergen molecules has been studied for adults [6,7] and evaluated in the BAMSE longitudinal birth cohort [33]. To the best of our knowledge, our study is the first to evaluate IgE sensitization and IgE levels specific to the complete panel of cat allergen molecules in clinically well-defined patients as well as to investigate the molecular profiles of IgE sensitization and cumulative allergen-specific IgE levels with phenotypes of cat allergy.

## 2. Results

### 2.1. Characterization of Cat Allergic Patients

**Figure 1:**
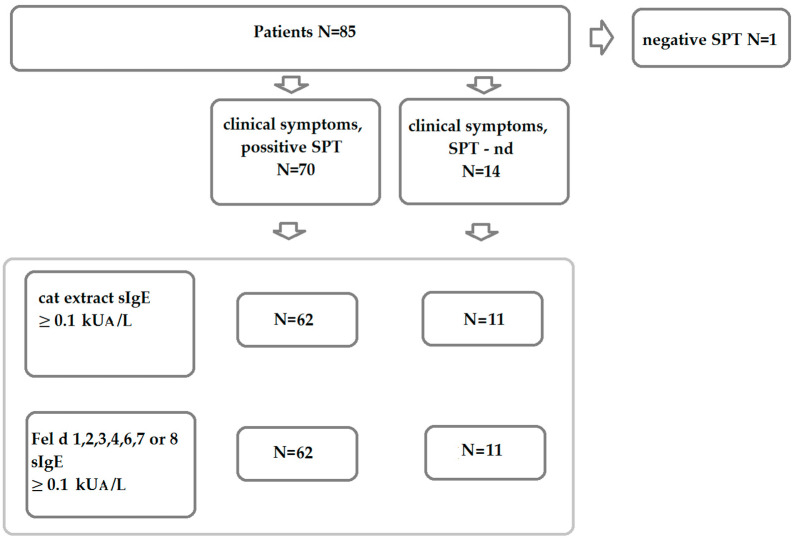
Consort diagram of the enrolment of patients.

Sera were collected from patients with clinical symptoms of cat allergy living in Moscow, the Moscow region, and Yekaterinburg. Figure 1 shows a consort diagram illustrating the enrolment of patients. From 85 patients with clinical symptoms to cat exposure, one patient with a negative skin test result to the cat dander extract was excluded from the study population. Sensitization to cat dander extract was confirmed in 70 of the 84 patients by skin testing; for 14 patients, no skin test results were available, but these patients were kept in the study because they exhibited documented allergic symptoms upon cat exposure. Thus, the study included 84 patients, 57 male and 27 female, with clinical symptoms of allergy upon cat exposure, such as asthma, rhinitis, conjunctivitis, and dermatitis, with a history of confirmed cat sensitization (Table S1). The patients were 18 to 53 years old, with a mean age of 27.19 years. Among them, 67 patients suffered from asthma, 81 patients had rhinitis, 70 patients had conjunctivitis, and 20 patients presented symptoms of dermatitis (Table 1 and Table S1).

Measurements of IgE antibody levels specific to cat dander extract and to seven purified cat allergen molecules (i.e., rFel d 1, nFel d 2, rFel d 3, rFel d 4, nFel d 6, rFel d 7, and rFel d 8) were performed in the two groups of patients, those with and without skin test results (Figure 1). We found that 62 out of the 70 patients and 11 out of the 14 patients had cat dander-specific IgE levels greater than or equal to 0.1 kU_A_/L, respectively (Table S1).

Among the 70 patients with positive skin test results, 62 showed specific IgE levels greater than or equal to 0.1 kUA/L for at least one of the tested allergen molecules, whereas 11 of the 14 patients without skin testing results were positive to at least one of the allergen molecules (Figure 1 and Table S1). Demographic and clinical characteristics of the 73 patients with cat allergen-specific IgE are shown in the lower part of Table 1 and in Table S1. The demographic and clinical features were comparable with those of the 84 patients with cat-related allergic symptoms (Table 1). Cumulative IgE levels greater than or equal to 0.1 kU_A_/L for cat allergen molecules were detected in 75 out of the 84 patients with cat-related allergic symptoms (Table S1). Interestingly, five skin test-positive patients had a negative result for IgE levels specific to the sum of allergen molecules (Table S1).

### 2.2. rFel d 1 Is the Major Cat Allergen but Cat Allergic Patients Are Also Sensitized to Several Other Cat Allergens

Table S1 shows the IgE levels specific to the cat allergen extract and cat allergen molecules for all 84 patients with cat-related allergic symptoms. Table 2 presents the mean IgE levels specific to the cat dander extract and to each of the seven tested allergen molecules in the 73 patients with cat allergen-specific IgE levels equal to or greater than 0.1 kU_A_/L (Figure 1). IgE sensitization to Fel d 1 was detected in 97.2% (*n* = 72) of the patients, and the mean level was 16.9 kU_A_/L (Table 2A). Thus, according to the set cutoff level, 2 out of 73 patients (i.e., patients 4 and 41) were identified as negative to rFel d 1, but they showed IgE reactivity to other cat allergen molecules (Table S1). nFel d 2-specific IgE was found in 30.1% of patients (*n* = 22), with a mean level of 2.7 kU_A_/L. rFel d 3-specific IgE was found in 50.6% of patients (*n* = 36), with a mean level of 2.18 kU_A_/L. rFel d 4-specific IgE was found in 52% of patients (*n* = 38), with a mean level of 5.69 kU_A_/L. nFel d 6-specific IgE was found in 32.8% of patients (*n* = 24), with a mean IgE level of 0.82 kU_A_/L. rFel d 7-specific IgE was found in 54.7% of patients (*n* = 40), with a mean level of 4.53 kU_A_/L. rFel d 8-specific IgE was found in 42.4% of patients (*n* = 32), with a mean level of 0.37 kU_A_/L (Table 2).

Three allergen molecules stood out as being recognized by more than 50% of the patients in our population (i.e., rFel d 3, rFel d 4, and rFel d 7). Accordingly, they may be considered as major allergens according to the definition in [34]. After rFel d 1, allergen-specific mean IgE levels were highest in the order rFel d 4 > rFel d 7 > nFel d 2 > rFel d 3 > nFel d 6 > rFel d 8 (Table 2).

### 2.3. The Cumulative Sum of IgE Levels Specific to Allergen Molecules Is Correlated with Cat Allergen Extract-Specific IgE Levels

**Figure 2:**
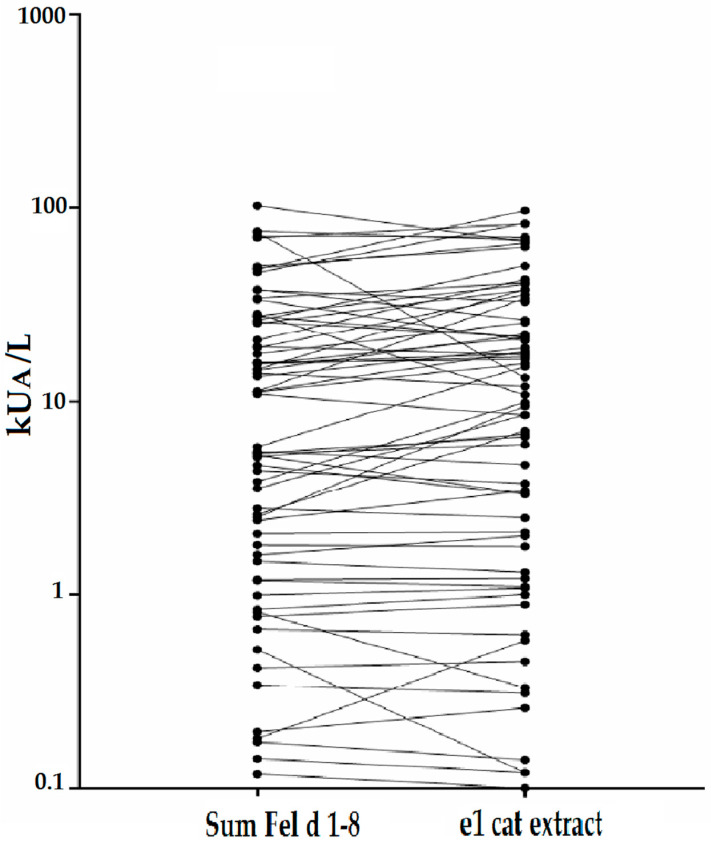
Correlation between the cumulative sum of Fel d 1–Fel d 8-specific IgE antibody levels and IgE levels to the cat allergen extract. Pearson’s correlation coefficient *r* = 0.822 (*p* < 0.01).

Cumulative allergen-specific IgE levels (i.e., the sum of IgE levels specific to the seven tested allergen molecules) were significantly correlated with IgE levels specific to cat allergen extract, indicating that the seven allergen molecules can be used to replace the cat allergen extract for measuring allergen-specific IgE levels (Figure 2). Specific IgE levels were comparable for the cat allergen extract and the sum of allergen molecules for most patients; however, certain patients showed higher IgE levels specific to cat allergen extract than to the cat allergen molecules or vice versa (Figure 2). Interestingly, when considering cumulative IgE levels greater than or equal to 0.1 kU_A_/L for the sum of allergen molecules, cat allergen-specific IgE reactivity was detected in 75 out of 84 patients with cat-related allergic symptoms. Thus, two more patients were detected with allergen molecules than with the cat allergen extract (Table S1). However, no cat allergen-specific IgE was detected in 9 out of 84 patients with cat-related allergic symptoms (Table S1). In five of the nine patients, a skin prick test with the cat allergen extract was performed, yielding positive results. In four patients, no skin testing was performed, but allergic symptoms related to cat exposure were recorded (Table S1).

### 2.4. Association of Symptoms and Phenotypes of Cat Allergy with Molecular IgE Recognition Profiles and Cumulative Allergen-Specific IgE Levels

**Figure 3:**
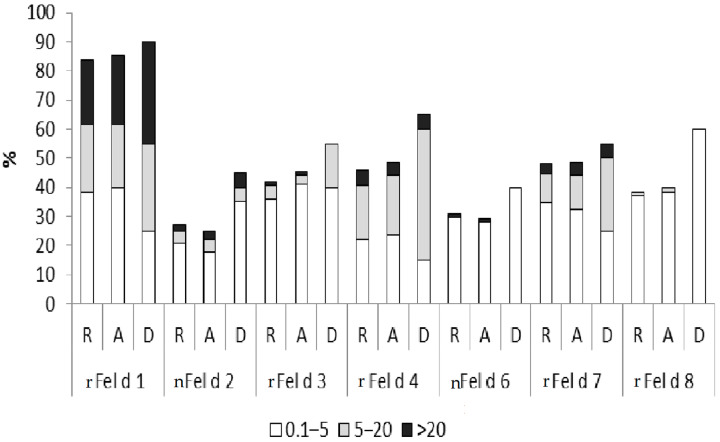
Prevalence and magnitude (*y*-axis: percentage of sensitized patients, measured in kU_A_/L, as indicated at the bottom) of IgE sensitization to cat allergen molecules in cat allergic patients (*n* = 73) with different symptoms (R: rhinitis; A: asthma; D: dermatitis).

**Figure 4:**
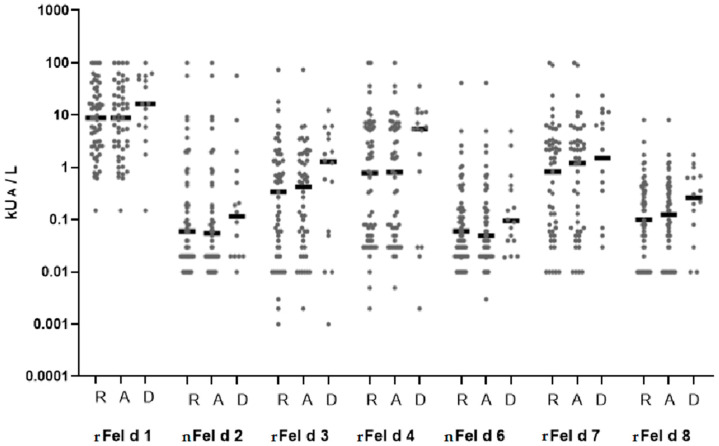
IgE levels (*y*-axis: sensitization, measured in kU_A_/L; median IgE levels are indicated by horizontal bars) specific to cat allergen molecules (Fel d 1–Fel d 8) in patients with different symptoms of cat allergy (R: rhinitis; A: asthma; D: dermatitis).

Figure 3 and Figure 4 show the prevalence of IgE recognition and allergen-specific IgE levels for each of the tested cat allergen molecules in patients with cat-related rhinitis, asthma, and dermatitis. Patients with cat-related skin symptoms showed a trend toward higher IgE levels and/or frequencies of sensitization to each of the tested allergen molecules compared with patients suffering from rhinitis or asthma, while there were no apparent differences between patients with rhinitis and asthma (Figure 3 and Figure 4).

**Figure 5:**
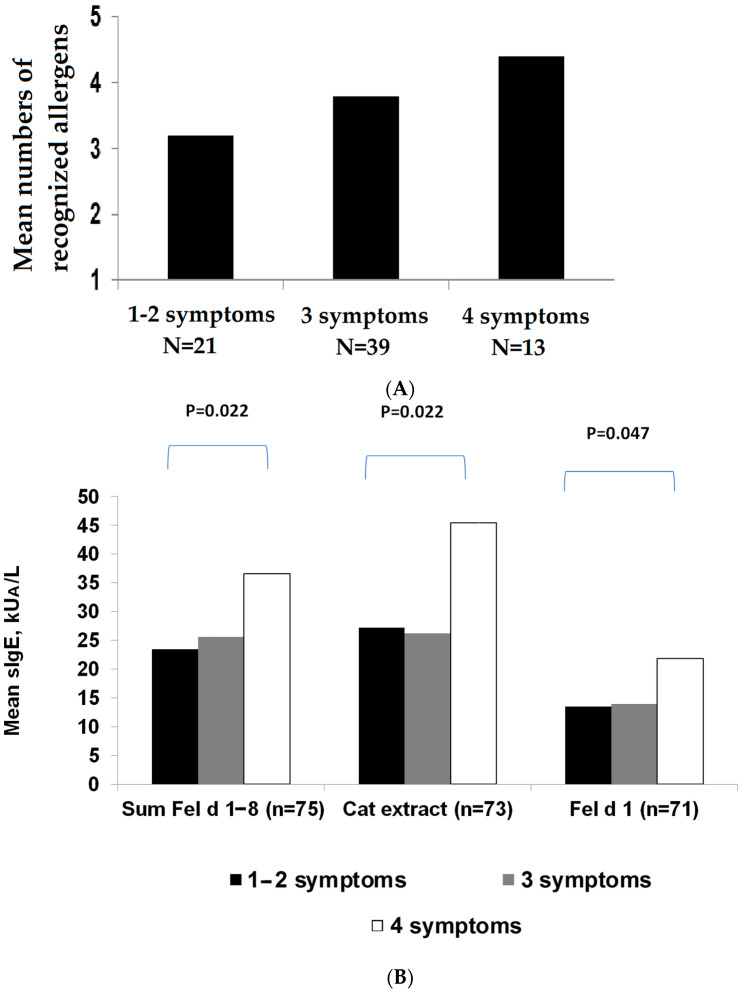
(**A**) Mean numbers of recognized allergens in patients suffering from 1–2, 3, or 4 cat-related allergic symptoms (asthma, rhinitis, conjunctivitis, and dermatitis); (**B**) mean levels of IgE (*y*-axis) specific to the sum of allergen molecules, the cat extract, and Fel d 1 (*x*-axis) in patients suffering from 1–2, 3, or 4 cat-related allergic symptoms (*x*-axis). Significant differences were calculated using the Mann–Whitney U test and are indicated for *p* ≤ 0.05 between the groups.

Next, we were interested in understanding if molecular allergen-specific IgE recognition profiles and cumulative allergen-specific IgE levels are associated with different phenotypes of cat allergy as defined by the number of different cat-related allergic symptoms. We found that 21 patients suffered from 1–2 symptoms of cat allergy, 39 patients suffered from 3 different symptoms of cat allergy, and 13 patients suffered from 4 different symptoms of cat allergy (Table S1). Figure 5A shows that the mean number of recognized allergens by patients with 1–2 symptoms was 3.2, the mean number of recognized allergens by patients with 3 symptoms was 3.82, and the mean number of recognized allergens by patients with 4 symptoms was 4.38 (Figure 5A). Although these differences were not significant, it can be seen in Figure 5A that a higher number of different allergic symptoms were recorded for a higher number of recognized allergen molecules.

When the sum of the IgE levels specific to allergen molecules, the IgE levels specific to the cat allergen extract, and the IgE levels specific to rFel d 1 were compared, it turned out that the allergen-specific IgE levels were significantly higher in patients with four symptoms compared with patients with 1–2 symptoms (Figure 5B). Interestingly, this difference was more pronounced for the sum of the IgE levels specific to allergen molecules and the cat extract than for IgE levels specific to rFel d 1 alone (Figure 5B).

## 3. Discussion

To the best of our knowledge, our study is the first to determine IgE reactivity profiles and IgE levels specific to the complete panel of purified cat allergen molecules (i.e., rFel d 1, nFel d 2, rFel d 3, rFel d 4, rFel d 7, and rFel d 8) as well as cross-reactive carbohydrate epitopes, as represented by nFel d 6, in a clinically well-characterized group of adult cat-allergic patients. Several relevant results were obtained. First, we found that the panel of tested allergen molecules seemed to resemble the majority of IgE epitopes present in natural cat allergen extracts. This aspect was studied by quantifying the cumulative sum of cat allergen molecule-specific IgE concentrations and by comparing it with allergen extract-specific IgE levels using a test system, allowing for their quantification, i.e., the ImmunoCAP system [35]. We found that there was a significant and high correlation between the cumulative sum of allergen-specific IgE and allergen extract-specific IgE, indicating that the allergen molecules indeed resemble much of the IgE epitope repertoire of the natural allergen source. However, in this context, it should be mentioned that specific IgE reactivity could not be detected in all patients with cat-related allergic symptoms. There are several possible explanations for this finding. The first possibility is that there are additional not yet discovered allergen molecules that are not represented by the panel of allergen molecules. However, the fact that two patients who were negative with the allergen extract showed IgE reactivity to the sum of allergen molecules speaks against this possibility (Table S1). It is also possible that allergen-specific IgE reactivity below 0.1 kU_A_/L may cause a clinically relevant sensitization. Lastly, it is possible that, in certain patients, allergen-specific IgE antibodies are mainly bound to effector cells via IgE receptors and, thus, cannot be detected by IgE serology. This explanation is not unlikely because all of the patients who were skin-tested without allergen-specific IgE in serum showed positive skin reactions. Support for this explanation comes from a study that showed that treatment of allergic subjects with the anti-IgE antibody omalizumab, which prevents IgE binding to IgE receptors, could indeed visualize new IgE specificities in the sera of treated patients [36]. At present, we have no definitive proof regarding the correctness of the given explanations because purified recombinant and natural allergen molecules produced under good manufacturing practice (GMP) conditions are not available for skin testing. However, this open question does not affect the other findings of our study that were obtained for the large majority of patients containing allergen-specific IgE in their serum.

The determinations of the molecular IgE reactivity profiles were performed in a representative cohort of clinically well-characterized patients for whom cat-related symptoms of allergy were carefully recorded by case history, so that they could be definitively attributed to cat exposure. The fact that we did not perform provocation studies by exposing patients under controlled conditions to cats to confirm the symptoms of rhinitis, asthma, conjunctivitis, and dermatitis may be considered as a limitation of our study, but this could only have been performed in a large prospective study. Regardless of this limitation, our study demonstrated unambiguously that, in addition to the major cat allergen, rFel d 1, which was recognized by 97% of the cat allergic patients, other cat allergen molecules are important. rFel d 3, rFel d 4, and rFel d 7 were recognized by the IgE antibodies of more than 50% of the study population; hence, they must be considered as major or mid-tier allergens according to the frequency of IgE recognition. It is understood that measuring allergen-specific IgE reactivity alone is not sufficient to demonstrate the clinical relevance of additional allergen molecules in cat allergic patients; however, this finding highlights that it will be important to investigate in depth the contribution of allergens other than Fel d 1 to symptoms of cat allergy by provocation testing or at least by studying their allergenic activity in surrogate assays for allergic inflammation using freshly collected blood samples for basophil activation testing. Our study does, however, provide indirect evidence that allergen molecules other than Fel d 1 are clinically important because we found that the significance of the correlation of IgE levels specific to the sum of allergen molecules with complex phenotypes of cat allergy was higher than that of Fel d 1-specific IgE levels. Furthermore, we noted a trend toward higher IgE levels for allergens other than rFel d 1 in patients with symptoms of cat-related dermatitis.

Our study, thus, indicates that, in addition to rFel d 1, rFel d 3, rFel d 4, and rFel d 7 must be considered as important cat allergens. Accordingly, it will be important to consider additional cat allergens for allergen-specific treatment by AIT or passive immunization therapy for cat allergy. Furthermore, our study demonstrates the importance of molecular IgE diagnosis for cat allergy and indicates that the cumulative sum of cat allergen-specific IgE could be a biomarker for identifying patients with complex phenotypes of cat allergy. Our findings are, thus, important for the molecular diagnosis of IgE sensitization to cat and the design of specific molecular immunotherapies for treatment and prevention of cat allergy.

## 4. Materials and Methods

### 4.1. Characterization of Cat-Allergic Patients

As a first step, all patients completed the standardized ISAAC questionnaire for obtaining first-line evidence for or against an allergic phenotype [37]. Subjects who were positive according to the ISAAC questionnaire were further investigated for the presence of allergic sensitization using standard diagnostic methods for allergy including anamnesis, skin prick test [38], and total and specific IgE determination according to a traditional diagnostic pathway [39]. At the time of testing, patients had not received any therapy. Skin prick tests for verification of sensitization to cat could be performed in 66 of the patients who reported allergic symptoms upon cat exposure using cat allergen extracts from Microgen (Moscow, Russia) or Stallergenes (Antony, France). Allergic symptoms upon cat exposure were classified regarding asthma according to the ATS/ERS criteria from 2014 [40], and they were classified regarding rhinitis according to GINA guidelines [41,42] and European guidelines for the classification of rhinitis [43,44]. Furthermore, guidelines for the assessment of conjunctivitis [45] and skin manifestations were recorded as recommended [46]. Particular attention was paid to the discrimination of atopic dermatitis and urticaria. Only patients with atopic dermatitis were included under the term “dermatitis”. The study was approved by the ethics committees of the institutional review boards of Sechenov University and Moscow-based institutions and by the Ural State Medical University; the study was conducted according to the Declaration of Helsinki. Each patient signed an informed consent form.

### 4.2. Expression and Purification of Allergens

Recombinant Fel d 1 was expressed in *Escherichia coli* and purified as previously described [26]. Purified natural Fel d 2 and Fel d 6 were purchased from Sigma (Vienna, Austria), while antibodies and assays were purchased from Rockland (Pikeville, MD, US). Synthetic genes, codon-optimized for *E. coli* expression coding for Fel d 3, Fel d 4, Fel d 7, and Fel d 8 with 3′ DNA coding for a C-terminal hexahistidine tail, were synthesized and inserted into the *Nde*I/*EcoR*I sites of plasmid pET27b (ATG, Merzhausen, Germany). The DNA sequences and their correct insertion into the plasmid were confirmed by automatic sequencing of both DNA strands (ATG). *E. coli* BL21 DE3 transformed with the plasmids expressing Fel d 3, Fel d 4, Fel d 7, and Fel d 8 were grown at 37 °C in an incubator in LB medium containing kanamycin (34 μg/mL) at 37 °C until they reached a cell density (OD_600 nm_) of 0.3–0.6. The expressions of rFel d 3 or rFel d 7 proteins were induced by the addition of 1 mM isopropyl-β-thiogalactopyranoside for 3 h at 37 °C. The cells were harvested by centrifugation at 10,000× *g* for 10 min and frozen. The pellets from a 400 mL culture were opened with three cycles of freezing and thawing, resuspended in 40 mL of 20 mM Tris-HCl (pH 7) and 10 mM imidazole, and then incubated overnight at 4 °C with stirring. After centrifugation, the pellet was discarded and the supernatants were subjected to nickel affinity purification under native conditions, as described in the Qiagen purification protocol (Quiagen, Hilden, Germany).

The expression of the rFel d 4 or rFel d 8 proteins were induced by the addition of 1 mM isopropyl-β-thiogalactopyranoside for 3 h at 37 °C. The cells were harvested by centrifugation at 10,000× *g* for 10 min and frozen. The pellets from a 400 mL culture were resuspended in 40 mL of 8 M urea, 10 mM Tris-HCl (pH 8), and 100 mM NaH_2_PO_4_; further homogenized with an Ultra-Turrax (Ika, Stauffen, Germany) on ice; and then incubated overnight at 4 °C under constant stirring. The supernatant was subjected to nickel affinity chromatography under denaturing conditions (Qiagen). Fractions containing pure protein were united and refolded by extensive stepwise dialysis against 2 mM HEPES (pH 8) in the case of Fel d 4 and against 150 mM NaCl, 1 mM EDTA, and 20 mM Tris-HCl (pH 8.5) in the case of Fel d 8; they were stored in PBS as soluble proteins at −20 °C until use. The purity of the recombinant proteins and natural allergens was analyzed by SDS-PAGE (Figure S1). The molecular weight of the allergens was determined by matrix-assisted laser desorption/ionization time-of-flight mass spectrometry, as previously described [47], and it was found to match the molecular mass predicted according to their sequences.

### 4.3. Quantification of Allergen-Specific IgE Levels

For the quantification of allergen-specific IgE levels, an ImmunoCAP Phadia 100 instrument (Thermo Fisher Scientific/Phadia, Uppsala, Sweden) was used according to the instructions by the manufacturer. Streptavidin ImmunoCAPs (o212 ImmunoCAP, Thermo Fisher Scientific/Phadia) were used to prepare ImmunoCAPs containing rFel d 1, nFel d 2, rFel d 3, rFel d 4, nFel d 6, rFel d 7, and rFel d 8. Cat allergen extract-specific IgE levels were determined using the commercial ImmunoCAP e1 (Thermo Fisher Scientific/Phadia). For preparation of the molecular ImmunoCAPs allergens with streptavidin o212 ImmunoCAPs, each allergen was dialyzed extensively against 0.1 M NaHCO_3_ and 1 M NaCl at 4 °C for at least 8 h. The allergen concentration was then determined, and biotin labeling was performed as previously described [48,49,50].

Allergen-specific IgE levels greater than or equal to 0.1 kU_A_/L were considered positive [43,51].

### 4.4. Statistical Analyses

SPSS Statistics version 15.0 and Graphpad Prism version 6.0 were used for statistical calculations. Significant differences were calculated using the Mann–Whitney U test.

## 5. Conclusions

Our study showed that the panel of cat allergens consisting of rFel d 1, nFel d 2, rFel d 3, rFel d 4, nFel d 6, rFel d 7, and rFel d 8 resembled the IgE epitopes of the allergen source. The sum of cumulative IgE levels specific to allergen molecules was best correlated with different phenotypes, whereby higher levels indicated more symptoms. Our study also indicated that allergens in addition to rFel d 1 are important and need to be considered for molecular diagnosis and the development of molecular forms of allergen-specific treatment.

## Figures and Tables

**Table 1:** Demographic and clinical characteristics of patients with clinical symptoms of cat allergy (asthma, rhinitis, conjunctivitis, and dermatitis) (upper part) and of patients with cat allergen-specific IgE antibodies (lower part).

Sex		Age Mean (Min–Max)	Symptoms Upon Cat Exposure			
m	f		Asthma	Rhinitis	Conjunctivitis	Dermatitis
57	27	27.19 (18–53)	67	81	70	20
Patients with specific IgE antibodies > 0.1 kU_A_/L (*N* = 73)						
50	23	26.71 (18–52)	59	70	61	19

**Table 2:** Serological characteristics of patients with cat allergen-specific IgE antibodies. (A) Ranges and mean values of allergen-specific IgE levels. (B) Numbers and percentages of patients with allergen-specific IgE antibodies.

A							
Cat extract kU_A_/L mean	rFel d 1 kU_A_/L mean	nFel d 2 kU_A_/L mean	rFel d 3 kU_A_/L mean	rFel d 4 kU_A_/L mean	nFel d 6 kU_A_/L mean	rFel d 7 kU_A_/L mean	rFel d 8 kU_A_/L mean
27.06 (0.12–100)	16.94 (0–100)	2.7 (0–100)	2.18 (0–73.8)	5.69 (0–100)	0.82 (0–41.3)	4.53 (0–100)	0.37 (0–8.14)
B							
Cat extract *N* %	rFel d 1 *N* %	nFel d 2 *N* %	rFel d 3 *N* %	rFel d 4 *N* %	nFel d 6 *N* %	rFel d 7 *N* %	rFel d 8 *N* %
*N* = 73 100%	*N* = 71 97.2%	*N* = 22 30.1%	*N* = 36 50.6%	*N* = 38 52%	*N* = 24 32.8%	*N* = 40 54.7%	*N* = 32 42.4%

## Data Availability

Data supporting the reported results will be made available upon reasonable request to the corresponding author.

## Supplementary Materials

The following supporting information can be downloaded at https://www.mdpi.com/article/10.3390/ijms23136984/s1.
