# Supplementary material for: Molecular Allergen-Specific IgE Recognition Profiles and Cumulative Specific IgE Levels Associated with Phenotypes of Cat Allergy
Source: Int J Mol Sci. 2022 Jun 23;23(13):6984. doi: 10.3390/ijms23136984 (PMC9266786; doi:10.3390/ijms23136984)

International Journal of *Molecular Sciences*

MDPI

Table S1. Demographic, clinical, and serological characterization of the population of cat allergic patients.

1–2 symptoms
3 symptoms
4 symptoms

ID	Sex	Age	Prick Test	Cat Allergy Cl. Symp.				Fel d 1 (kUA/L)	Fel d 2 (kUA/L)	Fel d 3 (kUA/L)	Fel d 4 (kUA/L)	Fel d 6 (kUA/L)	Fel d 7 (kUA/L)	Fel d 8 (kUA/L)	e1 Cat Extract (kUA/L)	Sum Fel d 1–8
				Asthma	Rhinitis	Conj.	Der-mat.									
Positive sIgE to cat extract and/or allergen molecules																
1	m	31	+	+	+	−	−	3.19	9.22	0.74	0.65	0.65	1.25	neg	17.7	15.79
2	f	25	+	+	+	+	+	57.2	neg	neg	neg	0.17	neg	neg	100	57.49
4	f	35	+	+	+	+	+	neg	neg	neg	neg	neg	neg	0.53	0.62	0.611
5	m	29	+	−	+	+	+	1.76	neg	neg	neg	neg	neg	neg	1.77	1.803
7	m	24	+	−	+	+	−	5.05	neg	neg	neg	neg	neg	neg	6.76	5.113
8	f	57	+	−	+	+	−	2.55	neg	neg	neg	neg	neg	neg	7.02	2.61
9	f	27	+	+	+	+	+	8.79	0.86	1.23	5.37	0.15	2.18	0.31	35.4	18.89
10	m	29	+	+	+	+	−	0.36	neg	neg	neg	neg	neg	neg	0.45	0.417
11	m	29	+	+	−	+	−	5.2	neg	0.12	neg	neg	neg	neg	6.53	5.4
12	f	27	+	−	+	+	−	14.2	<u>3.7</u>	0.7	2.55	0.99	3.42	0.47	49.9	26.03
14	m	21	+	+	+	+	+	16.7	neg	0.59	1.78	0.019	6.35	0.15	21.5	25.68
15	m	19	+	+	+	+	−	100	neg	0.14	0.14	0.14	90.3	neg	100	>100
16	m	19	+	−	+	+	+	0.56	0.19	neg	neg	neg	neg	neg	0.89	0.767
17	m	21	+	+	+	+	−	5.43	0.78	1.79	5.96	0.11	neg	0.45	22.2	14.52
18	m	20	+	+	+	+	−	1.6	neg	0.54	neg	neg	3.03	0.2	4.67	5.49
20	m	18	+	+	+	+	−	29	neg	2.1	10.1	1.07	5.6	0.45	65.7	48.4
21	m	20	+	+	+	+	−	3.12	neg	neg	neg	neg	0.32	neg	8.49	3.54
23	m	23	+	+	+	+	+	13.2	neg	neg	neg	neg	neg	neg	18.9	13.34
24	m	22	+	+	+	+	−	2.55	neg	0.77	1.59	neg	neg	0.36	5.97	5.34
25	m	20	+	−	+	+	−	42.4	0.19	18	100	0.17	0.17	3.08	100	>100
26	m	23	+	+	+	+	+	0.69	neg	neg	0.1	neg	neg	neg	1	0.84
27	m	24	+	+	+	+	+	6.46	neg	1.35	5.96	0.69	neg	0.2	37.8	14.68
29	m	22	+	+	+	+	−	0.62	neg	0.34	1.94	neg	1.18	0.23	3.74	4.35
30	f	32	+	+	+	+	−	100	100	73.8	100	41.3	100	8.14	100	>500
31	m	24	+	+	+	+	−	0.24	neg	0.08	0.1	0.1	0.11	neg	0.33	0.81
33	f	42	+	−	+	+	−	15.5	neg	neg	neg	neg	neg	neg	17	15.63
34	m	41	+	+	+	+	+	100	<u>56.6</u>	6.19	11.1	4.96	neg	1.19	100	>100
35	f	40	+	+	+	+	−	22.8	neg	neg	neg	neg	2.19	neg	37.5	25.18
36	f	27	+	−	+	+	−	1.78	neg	neg	neg	neg	0.13	neg	2.1	2.06
37	f	39	+	+	+	+	−	100	neg	0.18	neg	0.21	2.68	neg	100	>100
38	m	28	+	+	+	+	−	15.6	neg	0.66	1.47	neg	9.53	0.18	40.3	27.49
39	f	28	+	+	+	+	−	0.12	neg	neg	neg	neg	neg	neg	0.58	0.18
40	f	38	+	−	+	+	−	4.5	neg	1.34	2.48	0.11	1.92	0.47	8.43	10.86
41	f	29	+	−	+	+	−	neg	neg	neg	neg	neg	0.47	neg	0.12	0.51

42	m	21	+	+	+	+	−	4.74	1.97	0.27	0.78	neg	3.3	0.1	18.1	11.21
43	m	25	+	+	+	−	−	0.23	neg	neg	neg	neg	neg	0	0.31	0.34
45	m	24	+	+	+	+	+	16.3	8.09	1.8	5.1	0.37	5.46	0.64	26.2	37.76
46	m	24	+	+	+	+	−	47.3	neg	neg	neg	neg	2.34	neg	62.6	49.75
47	m	21	+	+	+	+	−	2.23	neg	neg	neg	neg	0.13	neg	3.44	2.43
48	m	23	+	+	+	−	−	0.15	neg	neg	neg	neg	neg	neg	0.26	0.195
49	m	18	+	+	+	−	−	10	neg	0.12	0.33	0.73	neg	neg	33.8	11.24
50	m	21	+	+	+	+	−	32.3	5.6	3.61	27.3	1.96	neg	0.49	70.3	71.3
51	m	18	+	+	+	+	−	8.77	neg	neg	neg	neg	2.3	neg	15.7	11.16
52	m	22	+	+	+	−	−	0.16	neg	neg	neg	neg	neg	neg	0.14	0.173
53	m	20	+	+	+	−	−	1.34	0.17	3.58	7.54	0.16	0.18	0.92	11.9	13.89
54	m	26	+	+	+	+	+	34.5	0.19	5.98	36.3	neg	23.8	1.74	66.7	102.6
55	m	22	+	+	+	+	+	62.8	0.14	0.54	5.52	neg	0.52	0.22	82.2	69.83
56	m	22	+	+	+	−	−	5.77	0.45	0.68	7.17	neg	1.52	0.27	16.5	15.9
57	m	25	+	+	+	−	−	24	neg	1.82	7.16	0.4	neg	0.53	40.9	34.04
58	m	19	+	+	+	+	−	1.5	neg	neg	neg	neg	neg	neg	2.01	1.61
60	m	20	+	+	+	+	−	0.66	0.94	neg	0.12	0.11	2.8	neg	3.31	4.65
61	m	18	+	+	+	+	−	0.11	neg	neg	neg	neg	neg	neg	0.12	0.14
62	f	27	+	+	+	+	−	21.1	neg	2.17	7.8	neg	5.92	0.44	32.5	37.51
63	m	20	+	+	+	+	−	0.79	neg	neg	neg	neg	neg	neg	1.1	1.18
64	m	18	+	+	+	−	−	2.56	neg	neg	neg	neg	neg	neg	2.5	2.79
65	m	20	+	+	+	+	−	16.3	0.1	0.1	0.36	neg	2.16	neg	17.4	19.7
66	m	18	+	+	+	+	+	45.2	0.51	neg	0.83	0.45	0.83	neg	96.6	47.95
67	f	38	+	+	+	+	−	0.74	neg	3. 22	1.5	neg	1.5	neg	9.86	3.83
68	m	22	+	+	+	+	−	1.03	neg	neg	neg	neg	neg	neg	1.3	1.49
69	m	23	+	+	+	+	−	42.1	2.18	neg	neg	0.47	neg	1.25	82.8	46.13
70	f	28	+	+	+	+	−	8.21	neg	1.15	2.95	neg	2.95	0.32	21.3	15.6
71	m	26	nd	+	+	+	−	13	neg	1.36	6.29	neg	6.29	0.4	21.3	27.36
73	m	19	nd	−	+	−	−	17.5	neg	neg	neg	neg	neg	neg	25.4	17.58
75	m	34	nd	+	+	+	−	2.49	neg	neg	neg	neg	neg	neg	9.46	2.51
78	m	34	nd	−	+	+	+	56.2	neg	4.41	13.1	0.1	13. 1	0.36	13.2	74.17
82	f	31	nd	+	+	−	+	47.9	neg	3.51	11.5	neg	11.5	1	68.2	75.5
83	m	33	nd	+	−	+	+	3.2	neg	2	11.1	neg	11.1	0.64	10.8	28.08
85	f	47	nd	+	+	+	−	0.82	neg	neg	neg	neg	neg	neg	1.08	0.99
87	f	22	nd	+	+	+	−	13.7	neg	0.51	3.12	neg	3.12	0.15	42.5	20.66
88	f	52	nd	+	+	+	+	0.15	1.99	neg	neg	2.61	0.36	neg	3.32	5.23
91	f	22	nd	−	+	+	−	3.08	2.06	neg	neg	0.42	neg	neg	15.1	15.1
95	f	52	nd	+	−	−	−	1	neg	neg	neg	neg	neg	neg	1.21	1.19
96	f	22	nd	−	+	+	+	5.97	0.21	12.4	7.02	0.07	7.02	0.6	20.6	33.29
Cumulative sIgE to allergen molecules equal to or greater than 0.1 kUA/L																
13	m	20	+	+	+	+	−	neg	neg	neg	neg	neg	neg	neg	nd	0.115
59	m	21	+	+	+	+	−	neg	neg	neg	neg	neg	neg	neg	nd	0.11
Negative sIgE																
3	f	29	+	−	+	+	−	neg	neg	neg	neg	neg	neg	neg	nd	neg
19	m	18	+	+	+	+	−	neg	neg	neg	neg	neg	neg	neg	nd	neg
22	m	18	+	+	+	+	−	neg	neg	neg	neg	neg	neg	neg	nd	neg

28	m	24	+	+	+	−	−	neg	neg	neg	neg	neg	neg	neg	nd	neg
32	f	62	nd	+	+	+	+	neg	neg	neg	neg	neg	neg	neg	nd	neg
44	m	53	+	+	+	+	−	neg	neg	neg	neg	neg	neg	neg	nd	neg
72	f	27	nd	−	+	−	−	neg	neg	neg	neg	neg	neg	neg	nd	neg
80	f	34	nd	+	+	+	−	neg	neg	neg	neg	neg	neg	neg	nd	neg
84	m	28	nd	+	+	+	−	neg	neg	neg	neg	neg	neg	neg	nd	neg

Figure S1. Coomassie blue-stained SDS PAGE containing a molecular weight marker (left) and aliquots of 3 µg of purified protein cat allergen molecules (rFel d 1, nFel d 2, rFel d 3, rFel d 4, rFel d 7, and rFel d 8).

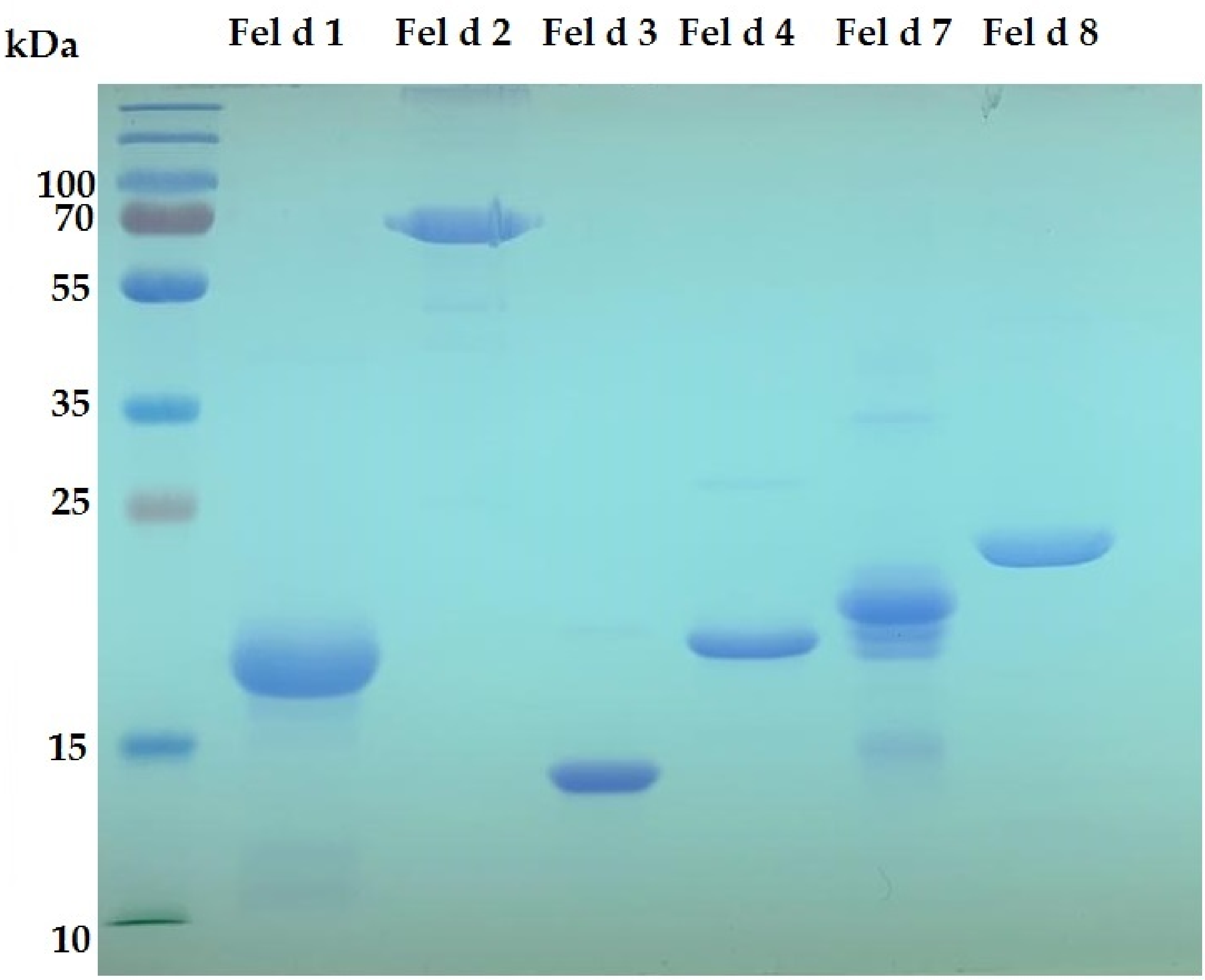

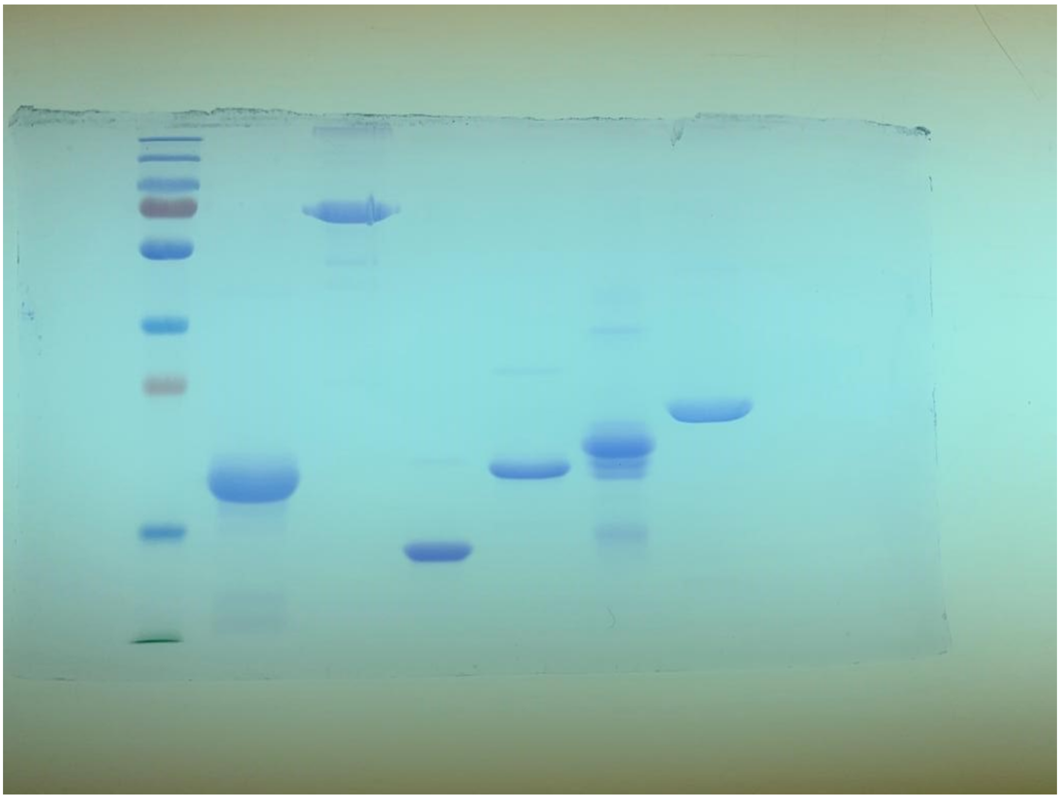

Supplement: Supplementary file 1 [file ijms-23-06984-s001.zip › ijms-1737103-supplementary.pdf]
